# Antimicrobial Action of Oleanolic Acid on *Listeria monocytogenes*, *Enterococcus faecium*, and *Enterococcus faecalis*

**DOI:** 10.1371/journal.pone.0118800

**Published:** 2015-03-10

**Authors:** Sejeong Kim, Heeyoung Lee, Soomin Lee, Yohan Yoon, Kyoung-Hee Choi

**Affiliations:** 1 Department of Food and Nutrition, Sookmyung Women's University, Seoul, Korea; 2 Department of Oral Microbiology, College of Dentistry, Wonkwang University, Iksan, Jeonbuk, South Korea; INSERM, FRANCE

## Abstract

This study investigated the antimicrobial action of oleanolic acid against *Listeria monocytogenes*, *Enterococcus faecium*, and *Enterococcus faecalis*. To determine the cytotoxicity of oleanolic acid, HEp-2 cells were incubated with oleanolic acid at 37^o^C. MICs (minimal inhibition concentrations) for *L*. *monocytogenes*, *E*. *faecium*, and *E*. *faecalis* were determined using two-fold microdilutions of oleanolic acid, and bacterial cell viability was then assessed by exposing the bacteria to oleanolic acid at 2 × MIC. To investigate the mode of antimicrobial action of oleanolic acid, we measured leakage of compounds absorbing at 280 nm, along with propidium iodide uptake. Scanning electron microscope (SEM) images were also analysed. The viability of HEp-2 cells decreased (*P* < 0.05) at oleanolic acid concentrations greater than 128 μg mL^-1^. The MICs were 16-32 μg mL^-1^ for *L*. *monocytogenes* and 32-64 μg mL^-1^ for *E*. *faecium* and *E*. *faecalis*, and bacterial cell viability decreased (*P* < 0.05) about 3-4 log CFU mL^-1^ after exposure to 2 × MIC of oleanolic acid. Leakage of 280 nm absorbing materials and propidium iodide uptake was higher in oleanolic acid –treated cells than in the control. The cell membrane was damaged in oleanolic acid-treated cells, but the control group had intact cell membrane in SEM images. The results indicate that oleanolic acid can kill *L*. *monocytogenes*, *E*. *faecium*, and *E*. *faecalis* by destroying the bacterial cell membrane.

## Introduction

Antibiotic-resistant bacteria have increased and spread globally due to improper use of antibiotics over recent decades, causing severe clinical problems. Development of novel therapeutics is therefore urgently required. Recently, various antimicrobial strategies have been introduced, and plant-derived compounds used as bactericides are receiving attention because of their low toxicity to human cell and ready availability.

Among them, oleanolic acid, an example of a pentacyclic triterpenoid originating from a number of medicinal plants, has antimicrobial activity against various bacterial pathogens [1, 2, 3, 4], and has various bioactivity [5, 6]. Its antibacterial activity is not much higher than traditionally-used antimicrobials, but the study on this antimicrobial is worthy because it is natural source product, and no resistance is found yet [7]. Our previous studies demonstrated that oleanolic acid inhibits the growth of *Listeria monocytogenes*, suggesting the potential usefulness of the compound as a food additive with anti-listerial activity [8]. In addition, it was shown that oleanolic acid inhibited the growth of Gram-positive bacteria, but this inhibition effect was weaker in Gram-negative bacteria [9].

Enterococci, Gram-positive bacteria, are considered to be part of the natural gut microflora in humans and animals that can be applied as starter cultures in food products, and as probiotics to improve the bacterial balance in the intestine [10, 11, 12]. They are also responsible for opportunistic infectious diseases, however, such as urinary tract, bloodstream, and nosocomial infections [13, 14]. Recently, enterococci have become more resistant to β-lactam-based antibiotics as well as aminoglycosides [15].

*L*. *monocytogenes* is well known as the causative agent of listeriosis, a food-borne illness with a high mortality rate [16]. This pathogen is able to survive under a variety of harsh environmental conditions, such as low pH, high salt, and low temperature [17]. Although sodium chloride, sodium nitrite, and a variety of other antimicrobials are used in processed foods to control the growth of *L*. *monocytogenes*, they have safety issues, making it necessary to develop new antimicrobials [18, 19]. In addition, because multidrug-resistant *L*. *monocytogenes* strains have emerged, and because most antibiotics cannot be given to pregnant patients with *L*. *monocytogenes* infections, we need to exploit natural therapeutic alternative to antibiotics [20, 21, 22].

Oleanolic acid is therefore potentially an excellent agent for eradicating the growth of *L*. *monocytogenes* in foods as well as clinical settings. To date, there has been extensive research on the activity of oleanolic acid against bacteria, but the studies have been mainly focused on measurements of the minimum inhibitory concentrations (MICs) of oleanolic acid against different microorganism. For various and suitable applications of antimicrobials, it is important to understand mode of these materials. Therefore, the aim of the present study was to investigate the mode of action of oleanolic acid against *L*. *monocytogenes*, *E*. *faecium*, and *E*. *faecalis*.

## Materials and Methods

### Preparation of oleanolic acid

A stock 5 mg mL^-1^ solution of oleanolic acid (Sigma, St. Louis, MO, USA) was freshly prepared in dimethyl sulfoxide (DMSO; Sigma). The solution was then diluted in brain heart infusion (BHI) broth (Difco, Becton, Dickinson and Company, Sparks, MD, USA) and tryptic soy broth (TSB; Difco, Becton, Dickinson and Company, Sparks, MD, USA) to 1,024 μg mL^-1^ for use in the experiments.

### Bacterial strains, cell line, and culture conditions

*L*. *monocytogenes* strains [NCCP10808 (ruminant brain isolate), NCCP10809 (human isolate), NCCP10920 (clinical isolate), NCCP10943 (rabbit isolate)], *E*. *faecium* strains [NCCP11193 (non-identified isolate)and NCCP10887 (cheese isolate)], and *E*. *faecalis* strains [KACC11304 (young chicken intestine isolate), NCCP10874 (non-identified isolate), and NCCP10886 (meat isolate)] were incubated in BHI broth and TSB for *L*. *monocytogenes*, and *E*. *faecium* and *E*. *faecalis*, respectively, for 24 h at 37°C. HEp-2 cells (human larynx epithelial cells, KCLB10023) were obtained from the Korean Cell Line Bank (Seoul, Korea) and cultured in Dulbecco’s Modified Eagles Medium (DMEM; Hyclone, Logan, UT, USA) supplemented with 10% fetal bovine serum (FBS; Hyclone) and 1% penicillin-streptomycin at 37°C in a 5% CO_2_ atmosphere.

### Cytotoxicity of oleanolic acid

HEp-2 cells (2 × 10^5^ mL^-1^) were plated in a 96-well microplate and incubated at 37°C for 48 h in a 5% CO_2_ atmosphere to allow cell attachment to the surface; the medium and unattached cells were discarded. Two-fold serial dilutions of oleanolic acid were prepared in DMEM plus 10% FBS and 1% penicillin-streptomycin, and the dilutions added to the wells. The plate was then incubated at 37°C for 48 h in a 5% CO_2_ atmosphere to determine cytotoxicity. 20 μL of MTT (3-(4,5-dimethylthiazol-2-yl)-2,5-diphenyltetrazolium bromide; Sigma) solution (5 mg mL^-1^) was added to each well, and the mixture was incubated at 37°C for a further 4 h in a CO_2_ atmosphere to allow the HEp-2 cells to metabolize MTT. The medium was then removed, the insoluble formazan crystals dissolved in 200 μL DMSO, and the absorbance measured at 550 nm using a spectrophotometer (Sunrise, Tecan, Austria). Data were reported as OD_550_ of oleanolic acid-treated cell.

### Determination of MICs and MBCs

MICs and MBCs of oleanolic acid were determined by the two-fold broth micro-dilution method [23]. All strains used in this study were grown overnight in each broth media at 37°C, followed by subculture in fresh each broth media and growth to logarithmic phase. An equal volume of the bacterial inoculum with an optical density of 0.01 at 600 nm was added to each well in a 96-well plate containing 100 μL of serially diluted oleanolic acid. After incubation for 24 h at 37°C, the MICs (minimal inhibition concentrations) were obtained by measuring the optical density at 600 nm with a spectrophotometer (Sunrise, Tecan, Austria). The lowest concentration of oleanolic acid at which no visible bacterial growth was observed was defined as the MIC for the bacteria. In addition, aliquots from the wells displaying no growth were spread on BHI agar plates for *L*. *monocytogenes* or tryptic soy agar (TSA) plate for *E*. *faecium* and *E*. *faecalis*, and the plates incubated at 37°C for 24 h to determine whether the reduction of bacterial growth in the wells was caused by bacteriostatic or bactericidal activity of oleanolic acid. The lowest concentration of oleanolic acid in the wells that gave reduction in bacterial growth on the agar plates by 99.9% compared to the initial bacterial inoculum was expressed as the MBC (minimal bactericidal concentration).

### Bacterial cell viability assay

Bacterial cell viability in the presence of oleanolic acid was determined by a slight modification to a previously described protocol [24]. One mL of the subcultured bacterial cells (OD_600_ = 0.7) of *L*. *monocytogenes* NCCP10943, *E*. *faecium* NCCP11193, and *E*. *faecalis* KACC11304 was resuspended in sterile saline and then added to 19 mL of 50 mM phosphate buffer (pH 7.1) with or without 2 × MIC of oleanolic acid [24]. Control was the cells without 2 × MIC of oleanolic acid, which was containing oleanolic acid-equivalent volume of DMSO. After 60 min incubation at 37°C, viable cell counts were taken for the suspensions on BHI agar plates for *L*. *monocytogenes* or TSA plates for *E*. *faecium* and *E*. *faecalis* and expressed as log CFU mL^-1^.

### Leakage of compounds absorbing at 280 nm

Bacterial cell suspensions prepared as for bacterial cell viability assays were used to measure the leakage to the supernatant of compounds absorbing at 280 nm, using a spectrophotometer (Spectra Max250, Molecular Devices, USA] [24]. Briefly, bacterial suspensions were prepared as for bacterial cell viability assay. One mL of the suspensions was treated with oleanolic acid or same volume of DMSO (control) for 60 min. After centrifugation (10,000ⅹg, 10 min), the absorbance of cell supernatant at 280 nm was determined. Release of compounds absorbing at 280 nm was expressed as a relative ratio of OD_280_ of oleanolic acid-treated cells to one of oleanolic acid-untreated cells.

### Propidium iodide uptake assay

A propidium iodide uptake assay was used to characterize the action of oleanolic acid on cell membrane permeability of the bacterial pathogens [24]. After 60 min incubation with or without oleanolic acid, 50 μL of suspension prepared as for the cell viability assay was added to 950 μL of phosphate buffer in FACS tubes stored on ice. Propidium iodide (Sigma) dissolved in sterile milliQ water was then added to the tube to a final concentration of 10 μg mL^-1^. The mixture was subjected to FACS analysis using a flow cytometry (BD FACScalibur, San Jose, CA, USA). The fraction of propidium iodide-stained cells was obtained using CellQuest Pro software (BD CellQuest, San Jose, CA, USA). The results were compared with one of DMSO-treated cells.

### Scanning electron microscope image analysis

The effect of oleanolic acid on the bacterial cell membrane was further investigated by scanning electron microscopy (SEM; JSM-7600F, JEOL Ltd., Tokyo, Japan). *L*. *monocytogenes* NCCP10943, *E*. *faecium* NCCP11193, and *E*. *faecalis* KACC11304 were cultured in 10 mL of BHI broth and TSB for *L*. *monocytogenes*, and *E*. *faecium* and *E*. *faecalis*, respectively, for 24 h at 37°C. The 0.1 mL portions of the cultures were subcultured overnight in 10 mL of each broth media containing a glass slide to which cells attached and grew. After overnight incubation, the attached cells were incubated with one mL of oleanolic acid in PBS (phosphate buffered saline) at 2 × MIC or an equal volume of DMSO for 1 h at 37°C [25]. Cells treated with DMSO (no oleanolic acid) were used as control. Incubated cells were pre-fixed in 1.8% glutaraldehyde solution (Sigma) for 30 min, followed by washing three times with distilled water. After post-fixing in 2% osmium tetroxide (Sigma) for 20 min, the bacterial cells were washed three times and then dehydrated in a graded series of ethanol concentrations (25, 50, 75, 90, and 100%). The cells were platinum-coated using a sputter coater (108 auto; Cressington Scientific Instruments Ltd., Watford, England), and examined by field emission SEM.

### Statistical analysis

This study was repeated with two samples in each repeat (n = 4). The OD values and bacterial cell counts (log CFU mL^-1^) at the various oleanolic acid concentrations were analysed by the mixed model procedure of SAS version 9.2 (SAS Institute, Cary, NC, USA). All least squares means comparisons among the interactions were performed by pairwise *t*-tests at alpha = 0.05.

## Results and Discussion

According to previous studies, oleanolic acid exhibits antimicrobial activity against many Gram-positive bacteria, including *L*. *monocytogenes*, *E*. *faecalis*, *E*. *faecium*, *Streptococcus mutans*, and *Streptococcus sanguis* [1, 2, 3, 4]. There was weaker antimicrobial effect against Gram-negative bacteria, however, including *Acinetobacter baumannii*, *Burkholderia thailandensis*, *Escherichia coli*, *Klebsiella pneumoniae*, and *Pseudomonas aeruginosa* [9]. This difference can be explained by the presence of the outer membrane of Gram-negative bacteria, which possesses multiple drug efflux pumps [2]. For explaining the antimicrobial action of natural antimicrobials, the poisonous effects on structure and function of membrane have generally been investigated [26, 27]. Kurek et al. [28] suggest that peptidoglycan metabolism is a cellular target of oleanolic and ursolic acid, and we hypothesized that oleanolic acid may cause death of Gram-positive bacteria by destroying bacterial cell membrane.

**Fig 1:**
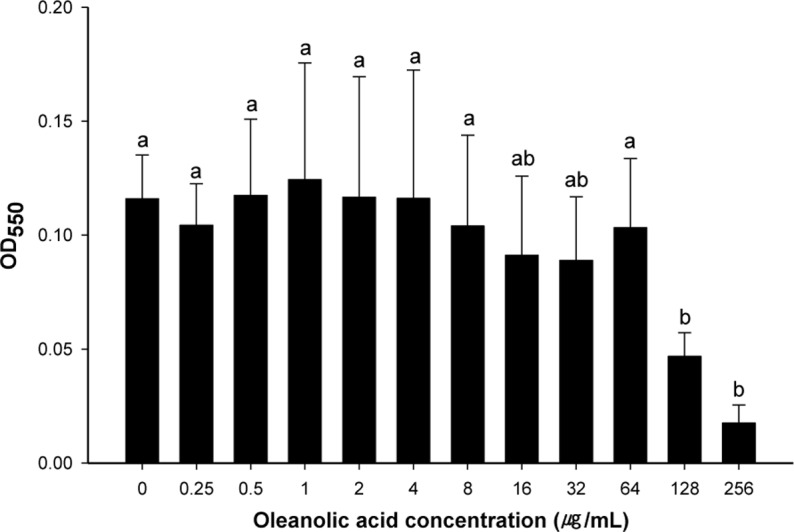
Cytotoxicity of oleanolic acid on HEp-2 cells. Cells were exposed to twofold-dilluted oleanolic acid (0–256 μg mL^-1^) for 48 hours. After exposure, viability of the cell was measured by MTT assay. a-b: means with different letters are significantly different (*P* < 0.05).

*In vitro* cell culture systems that retain similar marker enzymes in freshly isolated cells have been extensively used for assessing toxicity of antimicrobials in specific organs [29]. The cytotoxicity of oleanolic acid against HEp-2 cells was examined to determine if oleanolic acid can be used to inhibit the growth of pathogenic bacteria without having a cytotoxic effect on eukaryotic cells. Oleanolic acid caused no decrease in the viability of HEp-2 cells at concentrations up to 64 μg mL^-1^, but cell viability decreased at concentrations over 128 μg mL^-1^ (Fig. 1). This indicates that oleanolic acid is not cytotoxic at levels up to 64 μg mL^-1^. Although oleanolic acid can be consumed directly or indirectly by humans, it is important to know its effect on eukaryotic cells. Many other studies investigated the effect of oleanolic acid on the viability of eukaryotic cells, including leukemic cell line [30], normal liver cell, liver cancer cell line [31, 32] and kidney cell line [29]. The results in these studies indicated that oleanolic acid had no cytotoxicity on eukaryotic cells upto 45 μg mL^-1^. Fontanay et al. [4] also suggested that IC_50_ (50% inhibitory concentration) of oleanolic acid on HaCaT cell line were 52.4 μg mL^-1^. Taken together, the application of oleanolic acid for human may be appropriate.

The antimicrobial effects of oleanolic acid on *L*. *monocytogenes*, *E*. *faecium*, and *E*. *faecalis* were assessed by MIC and MBC. MIC is defined as the minimum concentration that allows no visible bacterial growth; MBC is the lowest concentration that kills bacteria by 99.9% [33]. Oleanolic acid inhibited the growth of *L*. *monocytogenes* strains at 16 μg mL^-1^ other than *L*. *monocytogenes* NCCP10808 that demonstrated no visible growth at 32 μg mL^-1^. Growth of *E*. *faecium* (NCCP11193 and NCCP10887) and *E*. *faecalis* (NCCP10874 and KACC11304) was inhibited at 32 μg mL^-1^, and MIC for *E*. *faecalis* NCCP10886 was 64 μg mL^-1^ (data not shown). As a result, the oleanolic acid had antimicrobial activity over the strains used in this study with strain variation. These MIC values are higher as observed in previous studies by Szakiel et al. [34] (15 μg mL^-1^ for *L*. *monocytogenes*) and Fontanay et al. [4] (8 μg mL^-1^ for *E*. *faecalis*). For vancomycin-resistant Enterococci, oleanolic acid had different antimicrobial activity according to strains, 8 μg mL^-1^ [2] or 256 μg mL^-1^ [4], and it was also concluded that this difference may be caused by strain variation of bacteria. The results for MBC showed that all strains tested were completely killed (99.9%) by oleanolic acid at > 256 μg mL^-1^. It is usually regarded as bactericidal if the MBC of antimicrobial agents is not exceeding four times of the MIC [35]. Thus, this shows that oleanolic acid has bacteriostatic but not bactericidal effects on *L*. *monocytogenes*, *E*. *faecium* and *E*. *faecalis*. Moreover, since 64 μg mL^-1^ is the maximum concentration of oleanolic acid showing no cytotoxicity, its use at less than 64 μg mL^-1^ would be expected to be bacteriostatic to *L*. *monocytogenes*, *E*. *faecium*, and *E*. *faecalis*.

**Fig 2:**
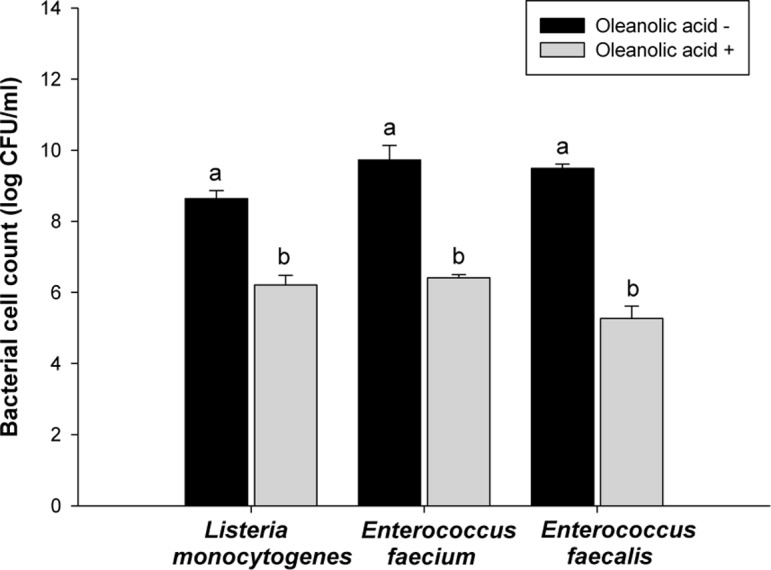
Bacterial cell viability. *Listeria monocytogenes*, *Enterococcus faecium*, and *Enterococcus faecalis* exposed to oleanolic acid at 2 × MIC. Viabilities of oleanolic acid-treated cells were compared with one of oleanolic acid-untreated cells. a-b: means with different letters are significantly different (*P* < 0.05).

A bacterial cell viability assay was conducted to measure the fraction of bacterial cells that were able to survive exposure to oleanolic acid. The results of bacterial cell viability assay showed short-time effect of oleanolic acid on the bacteria compared to MIC and MBC. After the bacterial cells were exposed to oleanolic acid for 60 min, *L*. *monocytogenes* viability decreased from 8.6 to 6.1 log CFU mL^-1^, (*p* < 0.05) (Fig. 2), and the viabilities of *E*. *faecium* and *E*. *faecalis* were reduced from 9.5 to 6.5 log CFU mL^-1^, and from 9.5 to 5.3 log CFU mL^-1^, respectively (*p* < 0.05) (Fig. 2). These results confirm the antibacterial effects of oleanolic acid on *L*. *monocytogenes*, *E*. *faecium* and *E*. *faecalis*.

**Fig 3:**
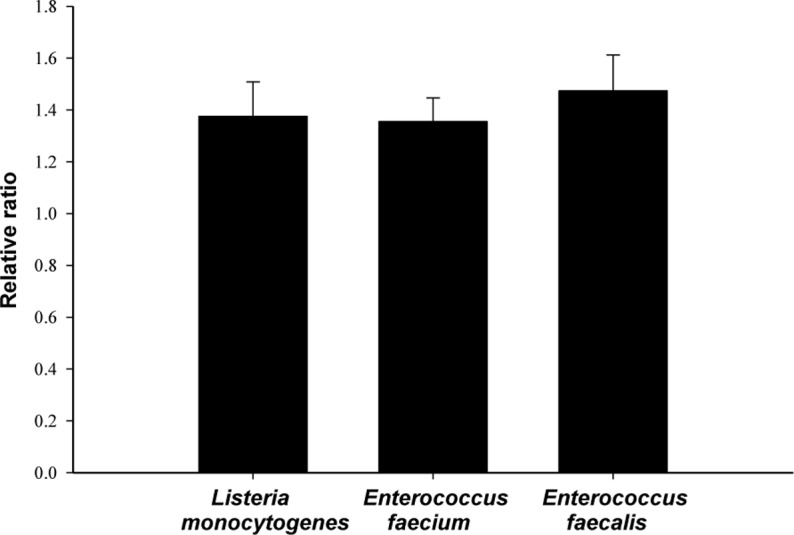
Leakage of material absorbing at 280 nm. After *Listeria monocytogenes*, *Enterococcus faecium*, and *Enterococcus faecalis* were exposed to oleanolic acid at 2 × MIC, leakage of material absorbing at 280nm was measured using spectrophotometer. The result was expressed as a relative ratio of OD_280_ of oleanolic acid-treated cells to the one of oleanolic acid-untreated cells.

We investigated potential membrane damage by measuring the leakage of bacterial cell contents caused by oleanolic acid. The ratios of compounds absorbing at 280 nm released by bacterial cells treated with oleanolic acid at 2 × MIC relative to those released by untreated cells were 1.38, 1.36 and 1.47 for *L*. *monocytogenes*, *E*. *faecium*, and *E*. *faecalis*, respectively (Fig. 3). Leakage of UV absorbing material is one of indicator presenting cell lysis [36]. This leakage of compounds absorbing at 280 nm is related to the loss of cell proteins that are normally retained by the cell membrane. These results indicate that the bacterial cell membrane is damaged by oleanolic acid.

**Fig 4:**
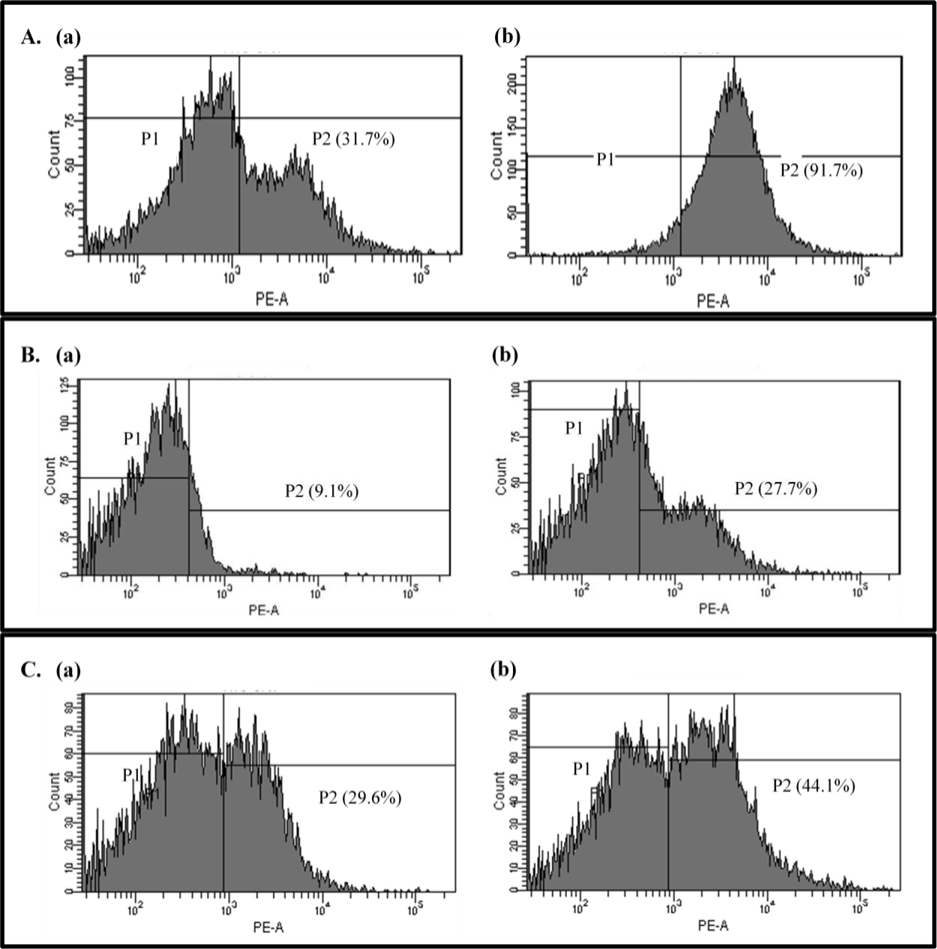
Propidium iodide uptake. Uptake of propidium iodide by untreated (a) or oleanolic acid-treated (2 × MIC) bacterial cells (b): *Listeria monocytogenes* (A), *Enterococcus faecium* (B), and *Enterococcus faecalis* (C). The area (P2) indicates the ratio of propidium iodide uptake cells.

To further investigate bacterial cell membrane damage, propidium iodide uptake was measured. Propidium iodide binds non-specifically to DNA and emits fluorescence, but due to its large size and negative charge, it cannot pass through intact membranes, fluorescence, therefore, indicates that the bacterial cell membrane is damaged and has become permeable. X-axis of the graph indicates level of emission. The more propidium iodide binds to DNA, the peaks are placed in the more right side of X-axis. The area in Fig. 4 indicates the ratio of propidium iodide uptake cells. After oleanolic acid treatment, fluorescent *L*. *monocytogenes* cells increased from 31.7% to 91.7%. Similarly, for *E*. *faecium*, the fraction of membrane-damaged cells went from 9.1% to 27.7%, and for *E*. *faecalis*, the percentage increased from 29.6% to 44.1% (Fig. 4). These results further support the idea that oleanolic acid destroys the cell membranes of Gram-positive bacteria.

**Fig 5:**
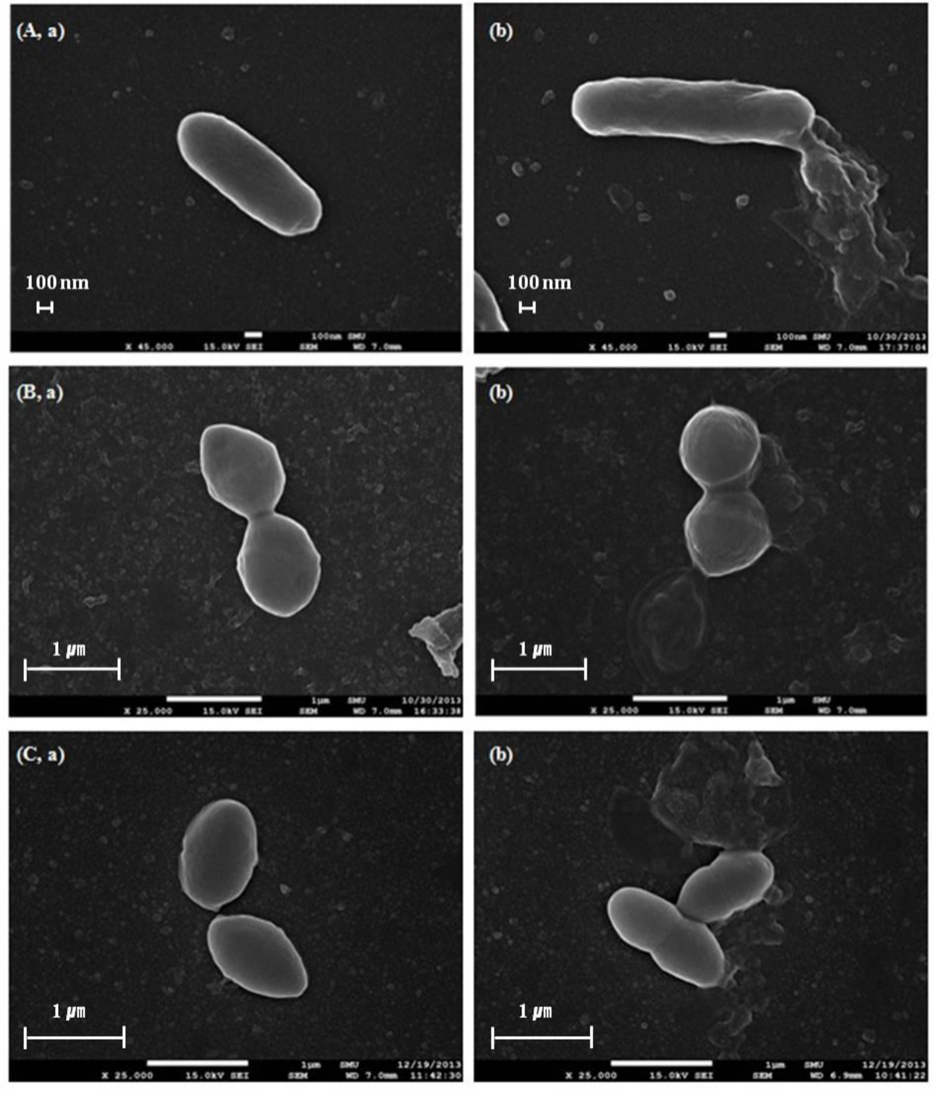
Scanning electron microscope images. After exposed to oleanolic acid at 2 × MIC, untreated (a) and oleanolic acid-treated bacterial cells (b): *Listeria monocytogenes* (A), *Enterococcus faecium* (B), and *Enterococcus faecalis* (C) were examined by SEM.

Bacterial cell damage by oleanolic acid was also visually confirmed by SEM. Fig. 5 shows SEM images of *L*. *monocytogenes*, *E*. *faecium*, and *E*. *faecalis* after oleanolic acid-treatment at 2 × MIC. Bacterial cells treated with oleanolic acid at 2 × MIC level showed changes in membrane shape in comparison to the control (DMSO-treated). The membranes of oleanolic acid-treated bacterial cells burst, whereas untreated cells had intact cell membranes (Fig. 5). This result directly confirms the implications of the cell leakage and propidium iodide uptake experiments that the antimicrobial activity of oleanolic acid against *L*. *monocytogenes*, *E*. *faecium* and *E*. *faecalis* results from damage to the cell membrane. Kurek et al. [28] found that oleanolic acid inhibited peptidoglycan turnover and influenced the muropeptides profiles, increasing autolysis of the cell wall. A study by Xia et al. [37] showed that oleanolic acid bound to peptidoglycan and teichoic acids, long anionic polymer threading through peptidoglycan. Recently, Kurek et al. [38] demonstrated that oleanolic acid bound to the peptidoglycan of the pathogen, which was influenced by teichoic acids. Hence, in our study oleanolic acid may bind to peptidoglycan of *L*. *monocytogenes*, *E*. *faecium* and *E*. *faecalis*, and this interaction was affected by teichoic acids, inhibiting peptidoglycan turnover.

In conclusion, oleanolic acid has antimicrobial effects on food-related bacterial pathogens, such as *L*. *monocytogenes*, *E*. *faecium* and *E*. *faecalis* by destroying the bacterial cell membrane, and the compound has low cytotoxicity on HEp-2 cells.

## References

[1] Kozai K, Suzuki J, Okada M, Nagasaka N. Effect of oleanolic-acid-cyclodextrin inclusion compounds on carries by *in vitro* experiment and rat-carries model. Microbios. 1999;97: 179–188. 10413873

[2] Horiuchi K, Shiota S, Hatano T, Yoshida T, Kuroda T, Tsuchiya T. Antibacterial activity of oleanolic acid from *Salvia officinalis* and related compounds on vancomycin-resistant enterococci. Biol Pharm Bull. 2007;30: 1147–1149. 17541170 10.1248/bpb.30.1147

[3] Jiménez AA, Meckes M, Torres JK, Luna HJ. Antimycobacterial triterpenoids from *Lantana hispida* (*Verbenaceae*). J Ethnopharmacol. 2007;111: 202–205. 17236730 10.1016/j.jep.2006.11.033

[4] Fontanay S, Grare M, Mayer J, Finance C, Duval RE. Ursolic, oleanolic and betulinic acids: antibacterial spectra and selectivity indexes. J Ethnopharmacol. 2008;120: 272–276. 10.1016/j.jep.2008.09.001 18835348

[5] Hung CY, Yen GC. Extraction and identification of antioxidative components of Hsian-tsao (*Mesona procumbens* Hemsl.). Food Sci Technol. 2001;34: 306–311.

[6] Kinjo J, Okawa M, Udayama M, Sohno Y, Hirakawa T, Shii Y, et al Hepatoprotective and hepatotoxic actions of oleanolic acid-type triterpenoidal glucuronoids on rat primary hepatocyte cultures. Chem Pharm Bull. 1999;47: 290–292. 10071859 10.1248/cpb.47.290

[7] Wolska KI, Grudniak AM, Fiecek B, Kraczkiewicz-Dowjat A, Kurek A. Antibacterial activity of oleanolic and ursolic acids and their derivatives. Cent Eur J Biol. 2010;5: 543–553.

[8] Yoon Y, Choi KH. Antimicrobial activity of oleanolic acid on *Listeria monocytogenes* under sublethal stresses of NaCl and pH. Korean J Food Sci Ani Resour. 2010;30: 717–721.

[9] Yoon Y, Choi KH. Identification of inhibitory effect on *Streptococcus mutans* by oleanolic acid. J Life Sci. 2010;20: 321–325.

[10] Gelsomino R, Vancanneyt M, Cogan TM, Condon S, Swings J. Source of enterococci in a farmhouse raw-milk cheese. Appl Environ Microbiol. 2002;68: 3560–3565. 12089042 10.1128/AEM.68.7.3560-3565.2002 PMC126786

[11] Saavedra L, Taranto MP, Sesma F, de Valdez GF. Homemade traditional cheeses for the isolation of probiotic *Enterococcus faecium* strains. Int J Food Microbiol. 2003;88: 241–245. 14596996 10.1016/s0168-1605(03)00186-7

[12] Canzek MA, Rogelj I, Perko B. Enterococci from Tolminc cheese: population structure, antibiotic susceptibility and incidence of virulence determinants. Int J Food Microbiol. 2005;102: 239–244. 15992623 10.1016/j.ijfoodmicro.2004.12.021

[13] Jett BD, Huycke MM, Gilmore MS. Virulence of enterococci. Clin Microbiol Rev. 1994;7: 462–478. 7834601 10.1128/cmr.7.4.462 PMC358337

[14] Hunt CP. The emergence of enterococci as a cause of nosocomial infection. Br J Biomed Sci. 1998;55: 149–156. 10198473

[15] Templer SP, Rohner P, Baumgartner A. Relation of *Enterococcus faecalis and Enterococcus faecium* isolates from foods and clinical specimens. J Food Prot. 2008;71: 2100–2104. 18939760 10.4315/0362-028x-71.10.2100

[16] Swaminathan B, Gerner-Smidt P. The epidemiology of human listeriosis. Microbes Infect. 2007;9: 1236–1243. 17720602 10.1016/j.micinf.2007.05.011

[17] Nuñez de Gonzalez MT, Keeton JT, Acuff GR, Ringer LJ, Lucia LM. Effectiveness of acidic calcium sulfate with propionic and lactic acid and lactates as postprocessing dipping solutions to control *Listeria monocytogenes* on frankfurters with or without potassium lactate and stored vacuum packaged at 4.5°C. J Food Prot. 2004;67: 915–921. 15151227 10.4315/0362-028x-67.5.915

[18] Mbandi E, Shelef LA. Enhanced antimicrobial effects of combination of lactate and diacetate on *Listeria monocytogenes* and *Salmonella* spp. in beef bologna. Int J Food Microbiol. 2002;76: 191–198. 12051475 10.1016/s0168-1605(02)00026-0

[19] Ramaswamy V, Cresence VM, Rejitha JS, Lekshmi MU, Dharsana KS, Prasad SP, et al *Listeria*-review of epidemiology and pathogenesis. J Microbiol Immunol Infect. 2007;40: 4–13. 17332901

[20] Ennaji H, Timinouni M, Ennaji MM, Hassar M, Cohen N. Characterization and antibiotic susceptibility of *Listeria monocytogenes* isolated from poultry and red meat in Morocco. Infect Drug Resist. 2008;1: 45–50. 21694879 10.2147/idr.s3632 PMC3108722

[21] Rahimi E, Yazdi F, Farzinezhadizadeh H. Prevalence and antimicrobial resistance of *Listeria* species isolated from different types of raw meat in Iran. J Food Prot. 2012;75: 2223–2227. 10.4315/0362-028X.JFP-11-565 23212021

[22] Adzitey F, Ali G, Huda N, Cogan T, Corry J. Prevalence, antibiotic resistance and genetic diversity of *Listeria monocytogenes* isolated from ducks, their rearing and processing environments in Penang, Malaysia. Food Control. 2013;32: 607–614.

[23] Mellegard H, Strand SP, Christense BE, Granum PE, Hardy SP. Antibacterial activity of chemically defined chitosans: Influence of molecular weight, degree of acetylation and test organism. Int J Food Microbiol. 2011;148: 48–54. 10.1016/j.ijfoodmicro.2011.04.023 21605923

[24] Reddy MV, Thota N, Sangwan PL, Malhotra P, Ali F, Khan IA, et al Novel bisstyryl derivatives of bakuchiol: Targeting oral cavity pathogens. Eur J Med Chem. 2010;45: 3125–3134. 10.1016/j.ejmech.2010.03.049 20427099

[25] Bereksi N, Gavini F, Benezech T, Faille C. Growth, morphology and surface properties of *Listeria monocytogenes* Scott A and LO28 under saline and acid environments. J Appl Microbiol. 2002;92: 556–565. 11872133 10.1046/j.1365-2672.2002.01564.x

[26] Cox SD, Mann CM, Markham JL, Bell HC, Gustafson JE, Warmington JR. et al The mode of antimicrobial action of the essential oil of *Melaleuca alternifolia* (tea tree oil). J Appl Microbiol. 2000;88: 170–175. 10735256 10.1046/j.1365-2672.2000.00943.x

[27] Tyagi AK, Bukvichki D, Gottardi D, Veljic M, Guerzoni ME, Malik A, et al. Antimicrobial potential and chemical characterization of serbian liverwort (*Porella arboris-vitae*): SEM and TEM observations. Evid Based Complement Alternat Med. 2013, Article ID 382927, 7 pages. 10.1155/2013/382927 PMC3556407 23365607

[28] Kurek A, Grudniak AM, Szwed M, Klicka A, Samluk L, Wolska KI, et al Oleanolic acid and ursolic acid affect peptidoglycan metabolism in *Listeria monocytogenes* . Anton Leeuw. 2010;97: 61–68. 10.1007/s10482-009-9388-6 19894138

[29] Madlala HP, MAsola B, Singh M, Musabayane CT. The effect of *Syzygium aromaticum*-derived oleanolic acid on kidney function of male Sprague-Dawley rats and on kidney and liver cell lines. Renal Failure. 2012;34: 767–776. 10.3109/0886022X.2012.678172 22512664

[30] Ovesna Z, Vachalkova A, Horvathova K, Tothova D. Pentacyclic triterpenoic acids: new chemoprotective compounds. Minireview. Neoplasma. 2004;51: 327–333. 15640935

[31] Wang X, Ye X, Chen HL, Bai H, Liang X, Zhang XD, et al Antioxidant activities of oleanolic acid *in vitro*: Possible role of Nrf2 and MAP kinases. Chem-Biol Interact. 2010;184: 328–337. 10.1016/j.cbi.2010.01.034 20100471

[32] Yan S, Huang C, Wu S, Yin M. Oleanolic acid and ursolic acid induce apoptois in four human liver cancer cell lines. Toxicol In Vitro. 2010;24: 842–848. 10.1016/j.tiv.2009.12.008 20005942

[33] Lee BL, Sachdeva M, Chambers HF. Effect of protein binding of daptomycin on MIC and antibacterial activity. Antimicrob Agents Chemother. 1991;35: 2505–2508. 1667253 10.1128/aac.35.12.2505 PMC245421

[34] Szakiel A, Ruszkowski D, Grudniak A, Kurek A, Wolsk KI, Doligalska M, et al Antibacterial and antiparasitic activity of oleanolic acid and its glycosides isolated from marigold (*Calendula officinalis*). Planta Med. 2008;74: 1709–1715. 10.1055/s-0028-1088315 18951335

[35] French GL. Bactericidal agents in the treatment of MRSA infections—the potential role of daptomycin. J Antimicrob Chemother. 2006;58: 1107–17. 17040922 10.1093/jac/dkl393

[36] Zhou K, Zhou W, Li P, Liu G, Zhang J, Dai Y. Mode of action of pentocin 31–1: an antilisteria bacteriocin produced by *Lactobacillus pentosus* from chinese traditional ham. Food Control. 2008;19: 817–822.

[37] Xia G, Kohler T, Peschel A. The wall teichoic acid and lipoteichoic acid polymers of *Staphylococcus aureus* . Int J Med Microbiol. 2010;300: 148–154. 10.1016/j.ijmm.2009.10.001 19896895

[38] Kurek A, Markowska K, Grudniak AM, Janiszowska W, Wolska KI. The Effect of Oleanolic and Ursolic Acids on the Hemolytic Properties and Biofilm Formation of *Listeria monocytogenes* . Pol J Microbiol. 2014;63: 21–25. 25033658

